# Transcriptome Analysis of Cyanide-Treated Rice Seedlings: Insights into Gene Functional Classifications

**DOI:** 10.3390/life12111701

**Published:** 2022-10-26

**Authors:** Cheng-Zhi Li, Yu-Juan Lin, Xiao-Zhang Yu

**Affiliations:** The Guangxi Key Laboratory of Theory & Technology for Environmental Pollution Control, College of Environmental Science & Engineering, Guilin University of Technology, Guilin 541004, China

**Keywords:** cyanide, COG classifications, differentially expressed genes, rice

## Abstract

Cyanide (CN^−^) pollution in agricultural systems can affect crop production. However, no data are available to describe the full picture of the responsive metabolic mechanisms of genes with known functions related to exogenous KCN exposure. In this study, we examined the transcriptome in rice seedlings exposed to potassium cyanide (KCN) using an Agilent 4×44K rice microarray to clarify the relationship between the differentially expressed genes (DEGs) and their function classifications. The number of DEGs (up-regulated genes/down-regulated genes) was 322/626 and 640/948 in the shoots and roots of CN^−^-treated rice seedlings, respectively. Functional predication demonstrated that a total of 534 and 837 DEGs in shoots and roots were assigned to 22 COG categories. Four common categories listed on the top five COG classifications were detected in both rice tissues: signal transduction mechanisms, carbohydrate transport and metabolism, post-translational modification, protein turnover and chaperones, and transcription. A comparison of DEGs aligned to the same COG classification demonstrated that the majority of up-regulated/down-regulated DEGs in rice tissues were significantly different, suggesting that responsive and regulatory mechanisms are tissue specific in CN^−^-treated rice seedlings. Additionally, fifteen DEGs were aligned to three different COG categories, implying their possible multiple functions in response to KCN stress. The results presented here provide insights into the novel responsive and regulatory mechanisms of KCN-responsive genes, and will serve as useful resources for further functional dissections of the physiological significance of specific genes activated in the exogenous KCN stress response in rice plants.

## 1. Introduction

Naturally, several metabolic pathways, such as the synthesis of ethylene, hydrolysis of cyanogenic compounds, and photorespiration and hydroxylamine from nitrate assimilation in plants, are able to generate the endogenous cyanide (CN^−^) [1,2,3], which regulates diverse physiological processes during plant growth and development [2,4]. It is known that the CN^−^ ligand has a strong binding affinity with Fe^3+^ ions in the protein cytochrome *c* oxidase in mitochondria to block the electron transport, eventually resulting in the functional disorder or repression of the respiration system [5]. In fact, the background of endogenous CN^−^ in plants is quite steady and does not cause any toxicity; this is because the dynamic balance between the input of CN^−^ and the assimilation of CN^−^ in the innate pool is driven by several enzymatic reactions in plants, including 1-amino-cyclopropane-1-carboxylic acid synthase (ACS, EC 4.1.1.14), aminocyclopropane-1-carboxylate oxidase (ACO, EC 1.14.17.4), β-cyanoalanine synthase (β-CAS, EC 4.4.1.9), sulfurtransferase (ST, EC 2.8.1.1), and nitrilase (NIT, EC 3.5.5.1) [3,6,7]. However, the main source of CN^−^ in the environment is anthropogenic. Mostly, CN^−^ is a frequently detected toxic chemical in the effluent of mining operations for gold extraction [8]. Other anthropogenic sources derived from electroplating, metal finishing and hardening, coke ovens, steel, and printed circuit board manufacturing are also introducing the flux of CN^−^ into the environment [9]. More than 100, 000 tons of CN^−^ generated annually can find their doors to enter various environmental matrixes [10]. Therefore, the discharge of CN^−^-containing effluent increases its ecological risk, and eventually poses a health issue to all living organisms [11]. Over the last two decades, many studies have investigated the mode of exogenous CN^−^ toxicity in plants, mainly based on physiological tests (e.g., the reduction in biomass growth and transpiration rate, and repression of pigment content and enzymatic activities [12,13]. For instance, KCN exposure at 2 mg CN/L caused a 50% inhibition of transpiration of willow cuttings (*Salix viminalis*). In comparison, a higher concentration of KCN at 20 mg CN/L killed the cuttings immediately [12]. Additionally, effective concentrations (ECs) of KCN in weeping willow cuttings (*Salix babylonica* L.) were also estimated using the normalized transpiration rate as a sensitive endpoint [14]. However, drawing a full picture of exogenous CN^−^-induced responses to plants has been difficult, particularly at the metabolic or gene-regulatory network scale.

Due to industrialization and population expansion, the agricultural system’s inability to afford various pollutants has become a public concern. As a result, more agricultural crops grown in multiple contaminated sites go to the market in different ways, which became a serious issue of food safety. Among various technologies developed to clarify these concerns, the characterization of specific gene functioning in different metabolic pathways offered more opportunities. In this regard, transcriptome analysis has been widely accepted to gain a global description of the gene expression profiles of plants in response to various environmental stimuli [15,16,17], and a variety of plant species were used, ranging from grass to crops. Rice, one of the most important staple crops, is the main energy food for half of the world’s population. It also acts as a suitable model plant for research because the full picture of the rice genome is well defined and sequenced. Previously, we used an Agilent rice microarray to investigate the molecular response of rice plants to potassium thiocyanate (KSCN) at different effective concentrations, and observed their different responsive and adaptive strategies [17]. Therefore, a deep insight into the metabolic or gene-regulatory network toward exogenous KCN exposure is necessary. In the present work, to obtain insights into the key COG classifications involved in the regulation and adaptation of rice plants to exogenous KCN exposure, we performed rice oligonucleotide arrays and verified them with real-time RT-PCR tests. The following analyses were conducted: (1) to determine the gene expression profile of rice seedlings in response to CN^−^ exposure using an Agilent 44K rice microarray; (2) to identify the differentially expressed genes (DEGs) in the roots and shoots of CN^−^-treated rice seedlings; (3) to construct co-expression network modules of DEGs by the STRING program; (4) to predicate and classify the DEGs activated in CN^−^-treated rice seedlings with the EggNOG 4.5.1 program. This comprehensive study on the expression profiles of genes in rice seedlings would be valuable for further exploration of physiological and biological mechanisms in response to CN^−^ exposure. 

## 2. Materials and Methods

### 2.1. Plant Materials and Exposure Regime

The preparation of rice (*Oryza sativa* L. cv. XZX 45) seedlings was identical to our previous work [7]. Briefly, rice seeds were sown in a small plastic cup with river sand and cultivated in an artificial climate box with constant conditions of light (20,000 lux), temperature (25 ± 0.5 ℃), and humidity (60 ± 2%). The modified ISO 8693 nutrient solution was used to support plant growth [7]. The main nitrogen source in the nutrient solution was potassium nitrate (KNO_3_) at 39.5 mg N/L. Sixteen days later, healthy seedlings of a similar size were selected, kept in an ion-cleaning buffer for 4 h, and then acclimated in the nutrient solution for 12 h. Finally, pre-treated seedlings were grown in the nutrient solution spiked with potassium cyanide (KCN) at 0 (control) and 1.0 mg CN/L for 2 d exposure. Potassium cyanide and the other chemicals used were all analytical grade. Each flask was covered with aluminum foil to minimize water loss and prevent algae growth. Each test was conducted in four independent replications.

### 2.2. RNA Extraction

After 2 days of exposure, the CN^—^treated rice seedlings were collected, rinsed with deionized water, and divided into the root and shoots. Treated and untreated plant tissues (0.2 g) were precisely weighted, immediately frozen in liquid nitrogen, and ground into fine powder. The total RNA was extracted from both the root and shoot of all rice samples by using an Ultrapure RNA Kit (CWBio, Taizhou, China). DNase I (CWBio, Taizhou, China) was used to remove genomic DNA contamination if any from the RNA extract. Then, the total RNA was purified with an RNeasy MinElute Cleanup Kit (Qiagen, Hilden, Germany).

### 2.3. Microarray Analysis

The Agilent 4X44K rice microarray with oligonucleotide 44,000 probes was used in this study. Shanghai Biotechnology Corporation (Shanghai, China) conducted microarray hybridization, washing, staining, scanning, and data processing. The data from the CN^−^ treatment were quantified as a fold change (FC) in comparison to the control (without CN^−^ treatment). After gaining all FC values, we deleted the invalid probes that were not expressed in the CN^−^ treatment according to the *IsGeneDetected* flag provided by AFE algorithms. After this filtering procedure, genes with defined function annotations were collected from the data of a CN^—^treated rice microarray analysis, which are known in the rice database RGAP (http://rice.plantbiology.msu.edu/analyses_search_blast.shtml (accessed on 12 February 2020). Additionally, the threshold for selection of the differentially expressed genes (DEGs) was set, *p* value < 0.05, and the fold change between treated and non-treated samples was >2.0 or <0.5.

### 2.4. PCR Verification 

A validation of the reliability of the data obtained from the rice microarray analysis was conducted with a qRT-PCR test. Twenty-five DEGs were randomly selected, and primers were designed using Primer 6.0 software. The same RNA samples described in RNA extraction were used. The RT-qPCR cycling conditions were as follows: (1) denaturation at 95 °C for 10 s, (2) annealing at 58 °C for 30 s, and (3) extension at 72 °C for 32 s. This cycle was imitated 40 times. The RT-qPCR analysis was executed using the 7500 Fast Real-Time PCR system (Applied Biosystems) (shanghai, China) and SYBR green chemistry [7]. Rice glyceraldehyde-3-phosphate dehydrogenase (LOC_Os08g03290.1) was used as the housekeeping gene. All the primer sequences of the selected genes, including the internal gene, are presented in Table S1. The standard 2^−ΔΔCT^ method was used to calculate the relative expression of the genes [18].

### 2.5. Classification of Gene Function 

The functional classification of DEGs in different tissues of CN^−^-treated rice seedlings was aligned to the Cluster of Orthologous Groups (COG) database using the EggNOG 4.5.1 (http://eggnogdb.embl.de (accessed on 28 August 2022).

### 2.6. Construction of Expression Network Modules

To establish functional modules of the genes, all the DEGs identified were uploaded to the Search Tool for the Retrieval of Interacting Genes (STRING) program (https://string-db.org/ (accessed on 14 September 2022), and the protein–protein interaction (PPI) networks (combined score > 0.4) were constructed. Finally, the modules (resolution = 0.8) with higher visualization were performed with the program Gephi 0.9.2.

## 3. Results

### 3.1. Identification of DEGs

**Figure 1:**
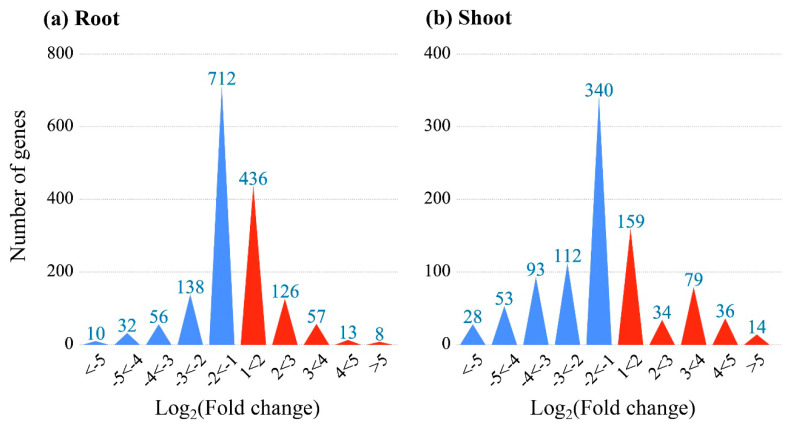
The DEGs in roots and shoots of CN^−^-treated rice seedlings.

To identify KCN response genes, the Agilent 4×44K rice microarray was used to analyze the expression profile and to select the DEGs in different tissues of rice seedlings. As shown in Figure 1, there were 948 DEGs identified in shoots with 322 up-regulated and 626 down-regulated genes. In comparison, 640 up-regulated and 948 down-regulated DEGs were identified in the roots of CN^−^-treated rice seedlings. 

### 3.2. PCR Verification of Microarray Data

**Figure 2:**
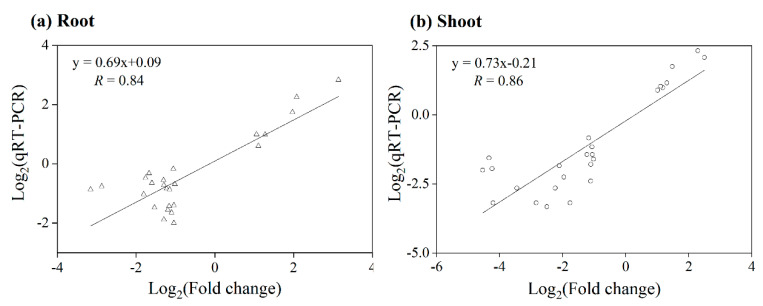
Correlation analysis of rice microarray data and qRT-PCR test for 25 DEGs in roots and shoots of CN^−^-treated rice seedlings, and linear regression with *R* value added.

Herein, 25 DEGs were selected for qRT-PCR tests to validate the reliability of the expression results obtained from the rice microarray under KCN treatments. A linear regression analysis demonstrated a significant correlation (root, *R* = 0.84; shoot, *R* = 0.86, *p* < 0.05) (Figure 2), judged by the Pearson correlation coefficient, showing a higher reliability of the rice microarray data.

### 3.3. Gene Function Analysis of DEGs

**Figure 3:**
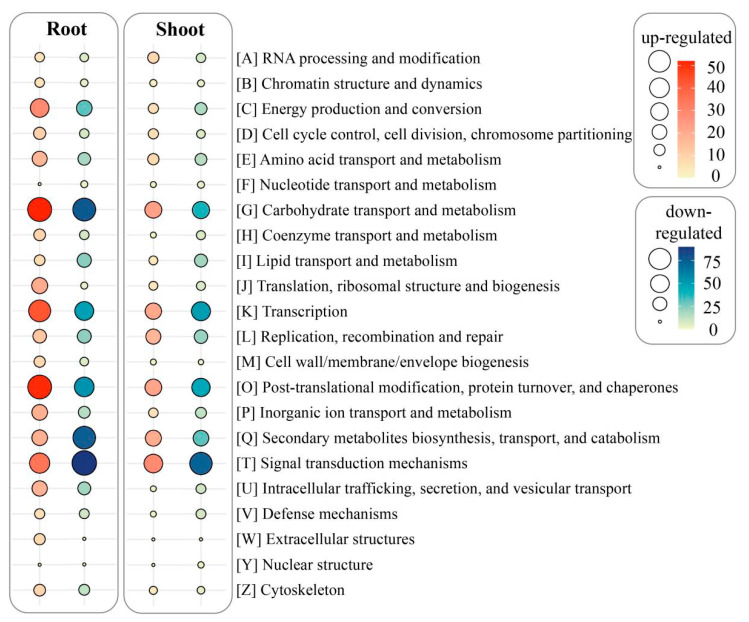
DEGs in roots and shoots of CN^−^-treated rice seedlings aligned to different COG classifications.

All the DEGs obtained were deposited in the COG database to conduct functional predication and classification. Overall, 534 DEGs (174 up-regulated/360 down-regulated) in the shoots and 837 DEGs (335 up-regulated/502 down-regulated) in the roots were assigned to 22 COG categories (Figure 3). We also noticed that 148 up-regulated/266 down-regulated DEGs in shoots were not mapped to any COG classifications. In comparison, there were 305 up-regulated/446 down-regulated DEGs in the roots without clear COG classifications. As shown in Figure 3, the top five COG classifications of the up-regulated/down-regulated DEGs in the shoots were mainly categorized into signal transduction mechanisms (29/73, 16.7%/20.3%), followed by carbohydrate transport and metabolism (23/39, 13.2%/10.8%); post-translational modification, protein turnover, and chaperones (22/46, 12.6%/12.8%); transcription (21/50, 12.1%/13.9%); and secondary metabolites biosynthesis, transport, and catabolism (20/31, 11.5%/8.6%). In contrast, the top five COG classifications of the up-regulated/down-regulated DEGs in the roots were chiefly associated with carbohydrate transport and metabolism (52/77, 15.5%/15.3%); post-translational modification, protein turnover, and chaperones (51/54, 15.2%/10.8%); transcription (43/49, 12.8%/9.8%); signal transduction mechanisms (35/90, 10.5%/17.9%); and energy production and conversion (29/31, 8.7%/6.2%).

### 3.4. Construction of Expression Network Modules of DEGs

**Figure 4:**
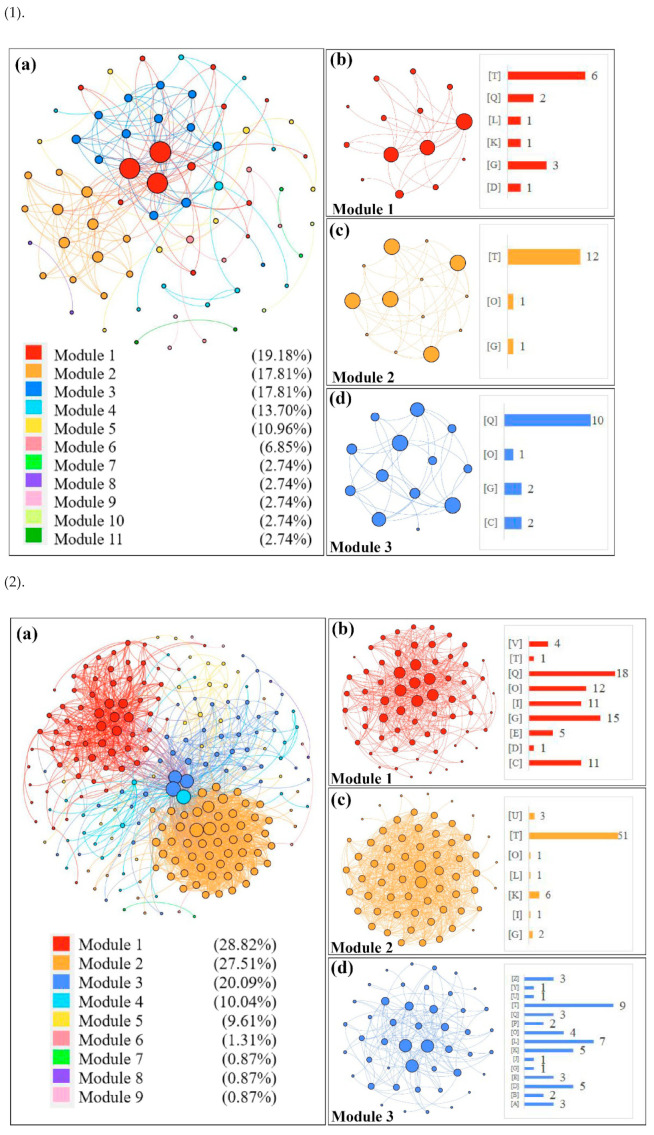

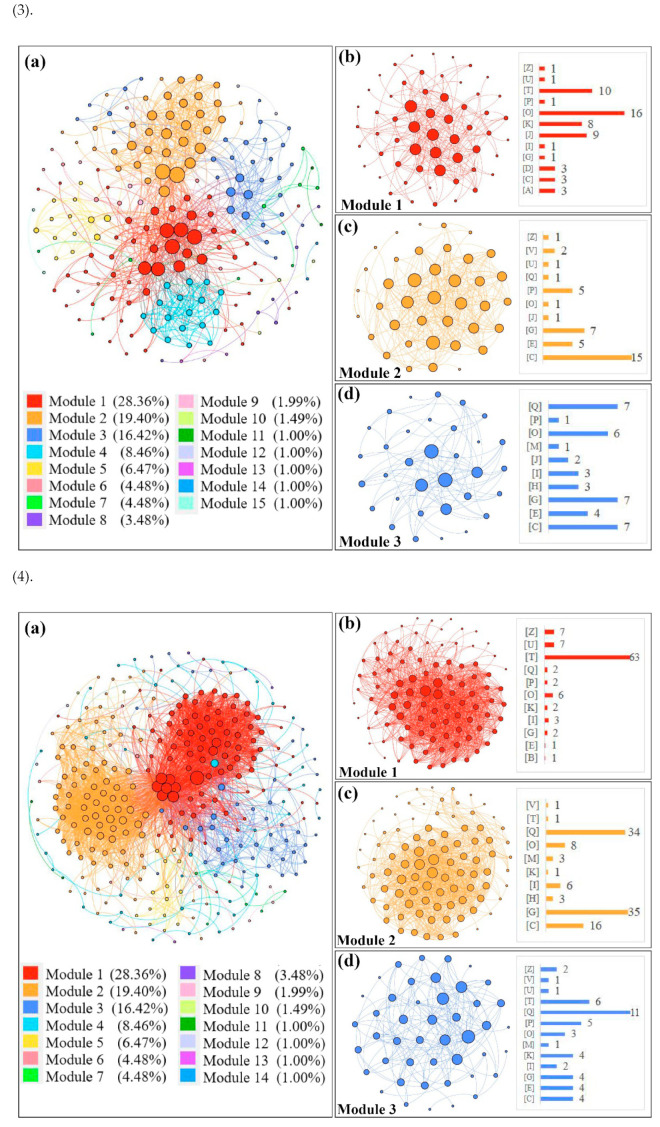
Construction of co-expression network modules of DEGs in CN^−^-treated rice seedlings by the STRING program. (**1**): modulation for up-regulated DEGs in shoots; (**2**): modulation for down-regulated DEGs in shoots; (**3**): modulation for up-regulated DEGs in roots; (**4**): modulation for down-regulated DEGs in roots. The letters in brackets refer to different COG classifications, as shown in Figure 3.

In order to group the functional modules of all the DEGs identified in different tissues of the CN^−^-treated rice seedlings, co-expression network modules were formed with the STRING program, and all functional modules were formed by the modularity calculation (Figure 4). Functional modules of the DEGs in the shoots are presented in Figure 4(1a) (up-regulated DEGs) and Figure 4(2a) (down-regulated DEGs), and three main modules with higher interaction contributions (>15%) are also shown in Figure 4(1b–d,2b–d), while the modularity of the DEGs in the roots is shown in Figure 4(3a) (up-regulated DEGs) and Figure 4(4a) (down-regulated DEGs). Detailed information of the up-regulated/down-regulated DEGs assigned to the top three modules from different tissues of the CN^−^-treated rice seedlings are given in Tables S2 and S3.

### 3.5. Comparative Analysis of DEGs

**Figure 5:**
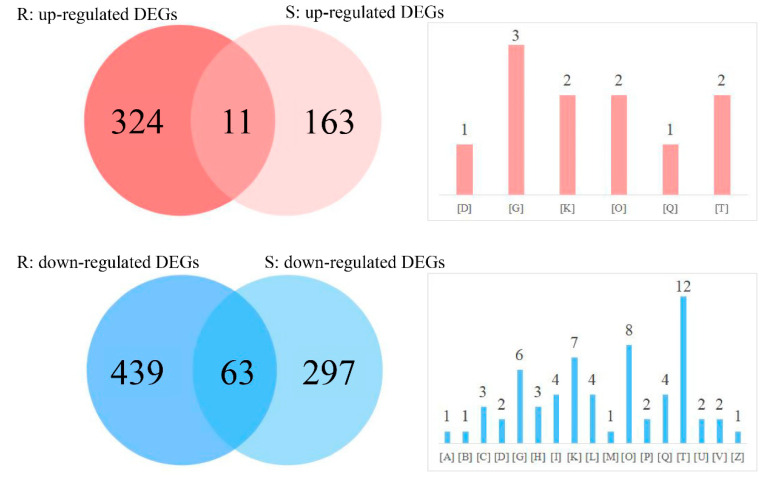
Comparative analysis of DEGs by a Venn diagram. The letters in brackets refer to different COG classification as shown in Figure 3.

A comparative analysis of the DEGs identified in different parts of the CN^−^-treated rice seedlings was conducted with a Venn diagram. Here, the DEGs with clear COG classifications were only used. As shown in Figure 5, 11 common up-regulated DEGs were obtained in the roots (3.3 %) and shoots (6.3%), wherein there were 63 common down-regulated DEGs obtained in the roots (12.5%) and shoots (17.5%). We noticed that the common up-regulated DEGs in both rice tissues were mapped into six different COG classifications. In comparison, the common down-regulated DEGs were aligned to 17 different COG classifications, suggesting that exogenous KCN exposure caused more negative changes to metabolic pathways, mainly through the signal transduction mechanisms. 

## 4. Discussion

### 4.1. Different Responses of Genes to KCN Exposure between Rice Tissues

It is evident that the assimilation potential of exogenous CN^−^ by various plants from different climate zones has been reported [12,13,19,20], in which all plants used, including both cyanogenic and non-cyanogenic species, are able to effectively remove the CN^−^ from different contaminated media. It has been reported that higher activities of β-cyanoalanine synthase were detected in cyanogenic plants than in non-cyanogenic plants [21]. However, our previous study did not support this conclusion. We observed that *Zea mays*, belonging to a cyanogenic family, had a relatively low metabolic capacity of exogenous KCN compared with other species tested [22]. We also noticed that the biological fate and distribution of CN^−^ in plant tissues was quite different between different parts of plants, in which more CN^−^ was detected in rice roots rather than shoots [7]. Roots are the first organ to come into contact with exogenous chemicals. An enzymatic and molecular analysis showed higher activities and a higher expression of β-cyanoalanine synthase in roots rather than shoots of CN^−^-treated rice seedlings [7]. In this study, a transcriptome analysis revealed that more down-regulated DEGs were detected in the roots of CN^−^-treated rice seedlings than shoots, suggesting that rice roots are more susceptible to exogenous CN^−^ exposure than shoots. Additionally, we noticed that many more up-regulated DEGs were identified in roots than shoots, and the ratio of the up-regulated DEGs to down-regulated DEGs in the roots (0.68) was higher than that in the shoots (0.51), implying that more physiological processes were positively activated in response to the KCN treatment. One possible explanation is that the dose of the CN^−^ used (1.0 mg CN/L) did not have a severe effect on the rice plants. In our previous work, the repression of exogenous CN^−^ at 1.0 mg CN/L on the plant growth of rice seedlings was negligible [7], most likely attributed to the involvement of non-toxic concentrations of CN^−^ in plants’ N nutrition through the assimilation pathway of β-cyanoalanine synthase [19,23]. Additionally, different nitrogen conditions altered the uptake and assimilation of exogenous KCN in rice seedlings, in which ammonium-fed rice showed a higher uptake and assimilation of KCN than nitrate-fed rice [7]. 

### 4.2. The Endogenous KCN Functions in Signaling Modulation

Most endogenous CN^−^ generated in plants is from ethylene biosynthesis [2], in which 1-amino-cyclopropane-1-carboxylic acid is converted into ET in the presence of aminocyclopropane-1-carboxylate oxidase, and CN^−^ is also produced as a concomitant simultaneously [1]. Assuredly, the endogenous CN^−^ functions in signaling modulation (e.g., phytohormone and ROS) in plants have been reported [2]. Although the dose used in this study (1.0 mg CN/L) did not cause inhibition on biomass growth and other visible toxicity symptoms at the physiological level, significant changes in the molecular level was evident. In this study, 29 and 35 up-regulated DEGs in the shoots and roots of CN^−^-treated rice seedlings were assigned in the classification of signal transduction mechanisms. In contrast, 73 (in shoots) and 90 (in roots) down-regulated DEGs were identified, implying the dual response to exogenous KCN exposure. This was most likely due to the presence of CN^−^ recovered in plant tissues. In fact, there are two degradation pathways to control the fate of CN^−^ in plant cells, namely the β-cyanoalanine synthase pathway and the sulfurtransferase pathway [3], and both pathways are activated in the assimilation of exogenous CN^−^ in plants. During exogenous KCN exposure, the production of endogenous CN^−^ through ethylene biosynthesis is still produced, judged by the up-regulation of aminocyclopropane-1-carboxylate oxidase [3]. The external feeding source of KCN was provided in the plant growth media. Although exogenous KCN did not accumulate in healthy plants [12], the residual CN^−^ in plant cells was significantly higher than the endogenous CN^−^ produced. Therefore, the dual roles of CN^−^ in plant cells are expected. 

### 4.3. Differences in DEGs Aligned to the Same Classification

Among the top five COG classifications obtained in this study, four common categories from all treatments existed. Here, we are interested in comparing the difference in up-regulated/down-regulated DEGs aligned to the same classification between the roots and shoots of CN^−^-treated rice seedlings. For instance, only three common up-regulated DEGs (LOC_Os04g51450.1, LOC_Os06g22980.1, and LOC_Os03g26620.1) aligned to the classification of carbohydrate transport and metabolism were identified, accounting for 5.8% and 13.0% of the total DEGs detected in the roots and shoots, respectively, wherein there were six down-regulated DEGs (LOC_Os01g46290.1, LOC_Os01g03360.1, LOC_Os02g37690.1, LOC_Os04g12600.1, LOC_Os10g06720.1, and LOC_Os05g40770.2) in common between the roots (7.8%) and shoots (15.4%). There were two common up-regulated DEGs (LOC_Os08g37730.1 and LOC_Os09g32948.1) and eight common down-regulated DEGs aligned to the classification of transcription (LOC_Os09g25060.1, LOC_Os05g50500.1, LOC_Os03g07450.1, LOC_Os05g45410.1, LOC_Os03g33012.1, LOC_Os02g57910.1, LOC_Os05g07010.2, and LOC_Os03g62870.3). Detailed information is given in Figures S1–S4. The results from this comparison indicated that more common down-regulated DEGs were detected from the same COG categories than up-regulated DEGs. For example, 6, 8, 8, and 12 common down-regulated DEGs were aligned to the classifications of carbohydrate transport and metabolism, transcription, post-translational modification, protein turnover and chaperones, and signal transduction mechanisms. In contrast, only 3, 2, 2, and 2 common up-regulated DEGs were detected, suggesting that responsive and regulatory mechanisms are quite tissue specific in CN^−^-treated rice seedlings.

### 4.4. Identification of DEGs Categorized in Different Classifications

In this study, we noticed that 15 DEGs were aligned to three different COG categories (Table 1), suggesting their multi-function activated in CN^−^-treated rice seedlings. Among these genes, 10 genes (e.g., LOC_Os01g08970.1, LOC_Os01g45350.1, LOC_Os12g12290.1, LOC_Os10g32080.1, LOC_Os06g23420.2, LOC_Os03g05070.1, LOC_Os10g32170.1, LOC_Os09g28470.1, LOC_Os07g09050.1, and LOC_Os12g33210.1) were positively related to KCN exposure, wherein five genes were down-regulated, namely LOC_Os04g54120.1, LOC_Os04g54120.1, LOC_Os02g57910.1, and LOC_Os07g03580.1. For instance, LOC_Os01g08970.1 was aligned to chromatin structure and dynamics; transcription; and replication, recombination, and repair classifications. Pandit et al. [24] observed that LOC_Os01g08970.1 functioned in transcription regulation as well as in DNA replication in the early developmental period of different varieties of rice plants in response to NaCl stress. Additionally, the involvement of LOC_Os01g08970.1 in transcriptional elongation was reported in rice suspension cells in the absence of the cell wall [25]. In this study, LOC_Os04g54120.1 was classified in the COG groups of transcription; replication, recombination, and repair; and signal transduction mechanisms, in which LOC_Os04g54120.1 regulated diverse cellular and biological processes in rice under multiple environmental stresses, such as water deficiency [26] and self-incompatibility and pathogen attack [27]. LOC_Os12g12290.1 and LOC_Os10g32170.1 are grouped in carbohydrate transport and metabolism classifications, cell wall/membrane/envelope biogenesis, and extracellular structures. Nivedita et al. [28] observed that a higher Na condition stimulated the expression of LOC_Os12g12290.1 to regulate ROS accumulation and cell wall integrity. Guo et al. [29] reported the involvement of LOC_Os10g32170.1 in hemicellulos synthesis in rice by integrating genomic and metabolomic frameworks. The role of the gene (LOC_Os10g32170.1) in allopathic interactions between rice and barnyardgrass was also observed in barnyardgrass co-cultured with rice [30]. Although the gene LOC_Os01g71256.1 was aligned to four different COG classifications, i.e., cell cycle control; cell division and chromosome partitioning; transcription, replication, recombination, and repair; and signal transduction mechanisms, there is not any literature to support its multi-function in rice plants in response to other environmental stimuli. We also noticed that 85 DEGs (8 up-regulated/19 down-regulated genes in shoots; 27 up-regulated/30 down-regulated genes in roots) were assigned to two different COG classifications. Further comprehensive studies on these specific genes would provide more powerful evidence to clarify their multiple roles in responses to different environmental stresses.

## 5. Conclusions

This systematic study provides a transcriptome analysis of CN^−^-treated rice seedlings using a rice microarray. Differential expression patterns of DEGs in different rice tissues indicate different responsive and regulatory mechanisms under exogenous KCN exposure. The functional predication of DEGs provides insights towards understanding their potential roles in responses to exogenous KCN exposure. Besides the findings presented here, our study provides a basic resource for future research on the roles of KCN-responsive genes activated in the specific pathway in rice plants.

## Figures and Tables

**Table 1:** DEGs in CN^−^-treated rice seedlings aligned to the same COG category.

R: Up-Regulated DEGs		R: Down-Regulated DEGs		S: Up-Regulated DEGs		S: Down-Regulated DEGs	
LOC_Os01g45350.1	[GMW]	LOC_Os01g08100.1	[CG]	LOC_Os01g08970.1	[BKL]	LOC_Os01g25189.5	[IT]
LOC_Os01g71256.1	[DKLT]	LOC_Os01g49240.1	[CG]	LOC_Os05g40990.1	[CH]	LOC_Os01g57470.1	[DZ]
LOC_Os02g34860.1	[DZ]	LOC_Os01g53420.1	[CG]	LOC_Os05g41660.1	[DO]	LOC_Os02g14680.1	[CG]
LOC_Os02g45980.1	[DZ]	LOC_Os01g53430.1	[CG]	LOC_Os05g45090.1	[CG]	LOC_Os02g37690.1	[CG]
LOC_Os02g47020.1	[TZ]	LOC_Os02g11640.1	[CG]	LOC_Os08g14810.1	[OT]	LOC_Os02g57910.1	[JKL]
LOC_Os03g05070.1	[GMW]	LOC_Os02g28900.1	[CG]	LOC_Os08g41890.1	[DZ]	LOC_Os03g55040.1	[CG]
LOC_Os03g52180.1	[IM]	LOC_Os02g33010.1	[CU]	LOC_Os09g19800.1	[EO]	LOC_Os03g62480.1	[CG]
LOC_Os03g55040.1	[CG]	LOC_Os02g37690.1	[CG]	LOC_Os11g25454.1	[CG]	LOC_Os04g20474.2	[CG]
LOC_Os03g55050.1	[CG]	LOC_Os02g41780.1	[EG]			LOC_Os04g46980.1	[CG]
LOC_Os04g12900.1	[CG]	LOC_Os02g42820.1	[TZ]			LOC_Os04g52050.1	[KT]
LOC_Os04g12960.1	[CG]	LOC_Os02g57910.1	[JKL]			LOC_Os04g54120.1	[KLT]
LOC_Os04g12970.1	[CG]	LOC_Os03g52170.1	[IM]			LOC_Os05g08480.1	[CG]
LOC_Os04g36720.1	[PQ]	LOC_Os04g25380.1	[CG]			LOC_Os05g12450.1	[CG]
LOC_Os04g47330.1	[BK]	LOC_Os04g35570.1	[DZ]			LOC_Os05g45110.1	[CG]
LOC_Os04g49430.1	[DZ]	LOC_Os04g57350.1	[TU]			LOC_Os06g05980.1	[EG]
LOC_Os06g23420.2	[GMW]	LOC_Os06g17020.1	[CG]			LOC_Os07g46950.3	[IQ]
LOC_Os07g09050.1	[GMW]	LOC_Os06g18790.1	[CG]			LOC_Os08g34780.1	[TZ]
LOC_Os07g47550.1	[CG]	LOC_Os06g30950.2	[EG]			LOC_Os09g34250.1	[CG]
LOC_Os09g19800.1	[EO]	LOC_Os07g03580.1	ADK			LOC_Os11g31770.1	[UY]
LOC_Os09g28470.1	[DTZ]	LOC_Os07g08050.1	[KL]				
LOC_Os10g20710.1	[TZ]	LOC_Os07g30620.1	[CG]				
LOC_Os10g32080.1	[GMW]	LOC_Os07g31960.1	[CG]				
LOC_Os10g32170.1	[GMW]	LOC_Os07g41060.1	[GM]				
LOC_Os12g03960.2	[EH]	LOC_Os07g46846.1	[IQ]				
LOC_Os12g04980.1	[DL]	LOC_Os08g01680.1	[BL]				
LOC_Os12g12290.1	[GMW]	LOC_Os08g41890.1	[DZ]				
LOC_Os12g33210.1	[KLT]	LOC_Os09g34230.1	[CG]				
		LOC_Os10g01134.1	[EO]				
		LOC_Os11g04860.1	[CG]				
		LOC_Os11g27329.3	[EO]				

The letters in brackets are the abbreviation for COG classifications, and the detailed information is given in Figure 3. The red background color refer to the genes assigned to three COG classifications and the blue background color refer to the gene assigned to four COG classifications.

## Data Availability

The authors acknowledge that the data presented in this study must be deposited and made publicly available in an acceptable repository before publication.

## Supplementary Materials

The following supporting information can be downloaded at: https://www.mdpi.com/article/10.3390/life12111701/s1. Table S1: Sequence of forward and reverse primers used in gene expression analysis; Table S2: DEGs in roots of CN^−^-treated rice seedlings assigned to top 3 modules; Table S3: DEGs in shoots of CN^−^-treated rice seedlings assigned to top 3 modules; Figure S1: DEGs aligned to the classification of carbohydrate transport and metabolism; Figure S2: DEGs aligned to the classification of transcription; Figure S3: DEGs aligned to the classification of post-translational modification, protein turnover, and chaperones; Figure S4: DEGs aligned to the classification of signal transduction mechanisms.
