# Supplementary material for: Transcriptome Analysis of Cyanide-Treated Rice Seedlings: Insights into Gene Functional Classifications
Source: Life (Basel). 2022 Oct 26;12(11):1701. doi: 10.3390/life12111701 (PMC9694641; doi:10.3390/life12111701)
Supplement: Supplementary file 1 [file life-12-01701-s001.zip › life-1987347-supplementary.pdf]

life

Supporting information for

Transcriptome Analysis of Cyanide-Treated Rice Seedlings: Insights into Gene Functional Classifications

Cheng-Zhi Li, Yu-Juan Lin and Xiao-Zhang Yu *

College of Environmental Science & Engineering, Guilin University of Technology, Guilin 541004, China
* Correspondence: xzyu@glut.edu.cn; Tel.: +86-7735897016

Supporting Information M1

1.1. RNA Extraction

Real-time quantitative PCR (RT-qPCR) was used to quantify expression levels of 25 DEGs identified in CN^{-}-treated rice seedlings. Total RNA was extracted from both root and shoot of all rice samples by using an Ultrapure RNA Kit (CWBio, Taizhou, China). DNase I (CWBio, Taizhou, China) was used to remove genomic DNA contamination if any from RNA extract. Then, the total RNA was purified with an RNeasy MinElute Cleanup Kit (Qiagen, Hilden, Germany). Each sample was prepared in four independent biological replicates.

1.2. PCR Analysis

All gene primer sequences are listed in Table S1. RT-qPCR cycling conditions were as follows: (1) denaturation at 95 °C for 10 s, (2) annealing at 58 °C for 30 s, and (3) extension at 72 °C for 32 s. This cycle was imitated 40 times. The RT-qPCR analysis was executed using the 7500 Fast Real-Time PCR system (Applied Biosystems) and SYBR green chemistry. Rice glyceraldehyde-3-phosphate dehydrogenase (LOC_Os08g03290.1) was selected as the house-keeping gene. The standard $2^{-\Delta\Delta CT}$ method was used to calculate the relative expression of each of the targeted genes.

Table S1. Sequence of forward and reverse primers used in gene expression analysis.

MSU ID	Primer Sequences(5′-3′)	Amplicon Size (bp)	Accession NO.
LOC_Os09g32948.1	F-CGGCCAAAGCATGACCAGA<br>R-ACCCGTGCCTTCAGTTTGAG	135	XM_015755338.1
LOC_Os04g08034.2	F-TGCAGTGAATACCGGATGTG<br>R-TGGCTCGGTGTCGAATAAAG	167	XM_026024798.1
LOC_Os02g36030.1	F-CTGTCATCGTCAACTAGTCACA<br>TTTCTCCTAAAGCACTCTCTCG	89	XM_015768639.2
LOC_Os03g51690.2	F-TCGCAATATCCTTTCCTCCG<br>R-CAGGTAGCTCTGTCTCTCCT	71	NM_001402246.1
LOC_Os06g22980.1	F-CGGGTACGACCACAACAAGA<br>R-GGCCTGGCAGTTGTAGATGT	124	XM_015787107.2
LOC_Os10g38740.1	F-CAAAGTGAGATGACATGACACG<br>R-CGATCAAGGAAACTCAATCGTG	121	NM_001404053.1
LOC_Os01g53240.1	F-TTCATCTACAACTACGCCGC<br>R-AATGTACGGCCATCGTCTTC	109	NM_001406101.1
LOC_Os01g09010.1	F-GTTCTTCCAGTTCGCCAAGG<br>R-GGAGACGTAGATGGACTCGTA	79	NM_001401471.1
LOC_Os07g44690.1	F-TGGTGGTGTCGATGGAGATG<br>R-CCTGACGAAGCTGGAGAAGT	233	NM_001403228.1
LOC_Os03g33012.1	F-TATGAAGGCAAGCACAGCCA<br>R-ACGTTGGCATTAGCGGAAGA	71	XM_026024276.1
LOC_Os01g50400.1	F-TCCAAGTGCTCAACGAGTCC<br>R-TCGGGATCAACACACTCTGC	142	XM_015783515.2
LOC_Os03g59740.1	F-CTGTGCTGCTTGTCTTTGCT<br>R-CCTCGTACAATCCCTCACCT	150	XM_026024224.1
LOC_Os11g14380.1	F-TCGAGTGGATGACACCGTTC<br>R-TAGTGACCGGACTTGCGATG	193	XM_015760390.2
LOC_Os12g18560.1	F-ATGGTGCTCATCGTCCTCC<br>R-AGTAGGAGGTGGAGTTGCAG	199	XM_015763615.2
LOC_Os01g49920.1	F-ACCATGAACTTGTCCGTGCA<br>R-GTTGCGGTGATGAGCCATTC	197	XM_026026747.1
LOC_Os01g11040.1	F-ATCTGGTGCGAAGTCCACTG<br>R-GGATTGTGCGGAATGAAGGC	144	NM_001404886.1
LOC_Os01g27360.1	F-CGCAAGTACAAGACGAGGGA<br>R-TGCACTCGTACACAATCGGT	133	NM_001401479.1
LOC_Os09g20220.1	F-CTCCTGGACTTGTGGGTGAG<br>R-CCTTCTTCTTCTCCGGCTCC	241	XM_015757106.2
LOC_Os02g26810.1	F-CTACCTCAACAAGTGCCGTG<br>R-TCCGCCTCAAGGATATGGTC	144	XM_015771093.2
LOC_Os04g12600.1	F-CAGCCGACTCCTTACCACAG<br>R-ATCTGGGTCAAGCGTAGCAC	283	XM_015779053.2
LOC_Os03g19420.1	F-GCGCTCTTCACCGACCTC<br>R-CTCCAGCTTGCTCAGGTTGA	231	XM_015775142.2
LOC_Os09g38790.1	F-CAGACTTCGTTCCCTATGGC<br>R-ATTAGAAGCGGGTGGAGGTA	243	XM_015756342.2
LOC_Os08g10320.1	F-AGCAACCACCAAGAACCGAT<br>R-GCCGTTGATGTAGGAGCAGT	197	XM_015794478.1
LOC_Os07g01820.5	F-ACTGGTGGAGAGGCAGAAGA	156	NM_001402986.1

	R-TGTGGTGGAAGAAGTGCCTG		
LOC_Os02g12380.1	F-TGATCCAGATGAAAGGCACG<br>R-AACCAAGATCGTCTGCCATC	194	XM_015772257.2
LOC_Os08g03290.1	F-GACAGCAGGTCGAGCATCTTC<br>R-CAGGCGACAAGCTTGACAAAG	74	NM_001403382.1

Table S2. DEGs in roots of CN^--treated rice seedlings assigned to the top 3 modules.

	**Number of DEGs in Roots**					
	**Up-Regulated**			**Down-Regulated**		
	**M 1**	**M 2**	**M 3**	**M 1**	**M 2**	**M 3**
[A] RNA processing and modification	3					
[B] Chromatin structure and dynamics				1		
[C] Energy production and conversion	3	15	7		16	4
[D] Cell cycle control, cell division, chromosome partitioning	3					
[E] Amino acid transport and metabolism		5	4	1		4
[F] Nucleotide transport and metabolism						
[G] Carbohydrate transport and metabolism	1	7	7	2	35	4
[H] Coenzyme transport and metabolism			3		3	
[I] Lipid transport and metabolism	1		3	3	6	2
[J] Translation, ribosomal structure, and biogenesis	9	1	2			
[K] Transcription	8			2	1	4
[L] Replication, recombination, and repair						
[M] Cell wall/membrane/envelope biogenesis			1		3	1
[O] Post-translational modification, protein turnover, and chaperones	16	1	6	6	8	3
[P] Inorganic ion transport and metabolism	1	5	1	2		5
[Q] Secondary metabolites biosynthesis, transport, and catabolism		1	7	2	34	11
[T] Signal transduction mechanisms	10			63	1	6
[U] Intracellular trafficking, secretion, and vesicular transport	1	1		7		1
[V] Defense mechanisms		2			1	1
[W] Extracellular structures						
[Y] Nuclear structure						
[Z] Cytoskeleton	1	1		7		2
**Total**	**57**	**39**	**41**	**96**	**108**	**48**

Table S3. DEGs in shoots of CN^{-}-treated rice seedlings assigned to the top 3 modules.

	**Number of DEGs in Shoots**					
	**Up-Regulated**			**Down-Regulated**		
	**M 1**	**M 2**	**M 3**	**M 1**	**M 2**	**M 3**
[A] RNA processing and modification						3
[B] Chromatin structure and dynamics						2
[C] Energy production and conversion			2	11		
[D] Cell cycle control, cell division, chromosome partitioning	1			1		5
[E] Amino acid transport and metabolism				5		3
[F] Nucleotide transport and metabolism						
[G] Carbohydrate transport and metabolism	3	1	2	15	2	1
[H] Coenzyme transport and metabolism						
[I] Lipid transport and metabolism				11	1	
[J] Translation, ribosomal structure, and biogenesis						1
[K] Transcription	1				6	5
[L] Replication, recombination, and repair	1				1	7
[M] Cell wall/membrane/envelope biogenesis						
[O] Post-translational modification, protein turnover, and chaperones		1	1	12	1	4
[P] Inorganic ion transport and metabolism						2
[Q] Secondary metabolites biosynthesis, transport, and catabolism	2		10	18		3
[T] Signal transduction mechanisms	6	12		1	51	9
[U] Intracellular trafficking, secretion, and vesicular transport					3	1
[V] Defense mechanisms				4		
[W] Extracellular structures						
[Y] Nuclear structure						1
[Z] Cytoskeleton						3
**Total**	**14**	**14**	**15**	**78**	**65**	**50**

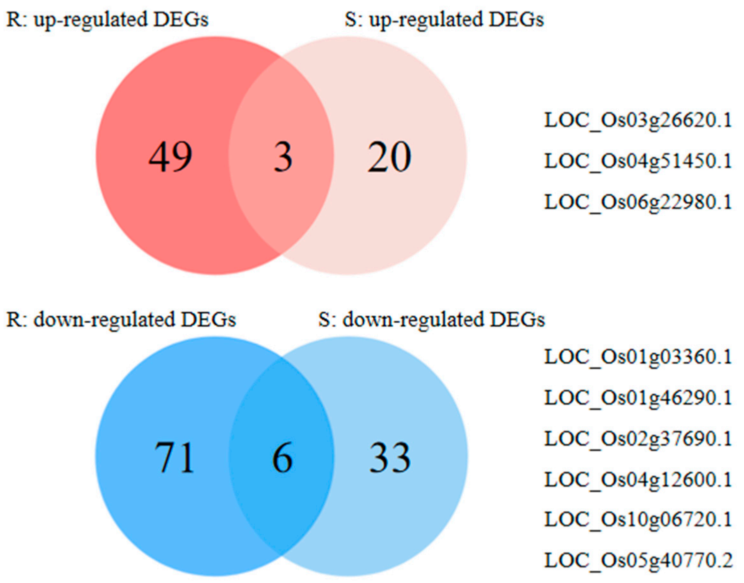

Figure S1. DEGs aligned to carbohydrate transport and metabolism classifications.

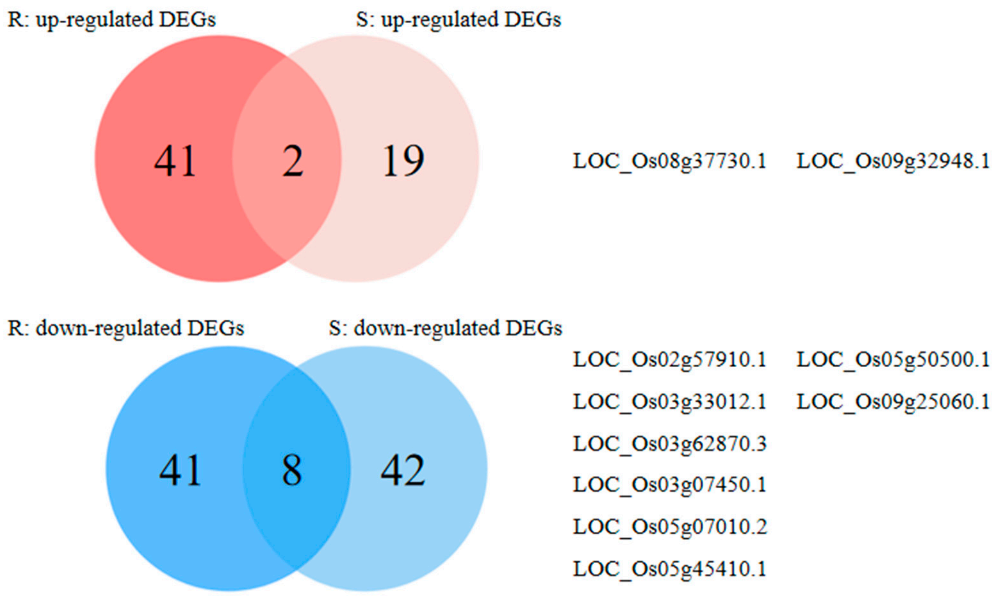

Figure S2. DEGs aligned to transcription classifications.

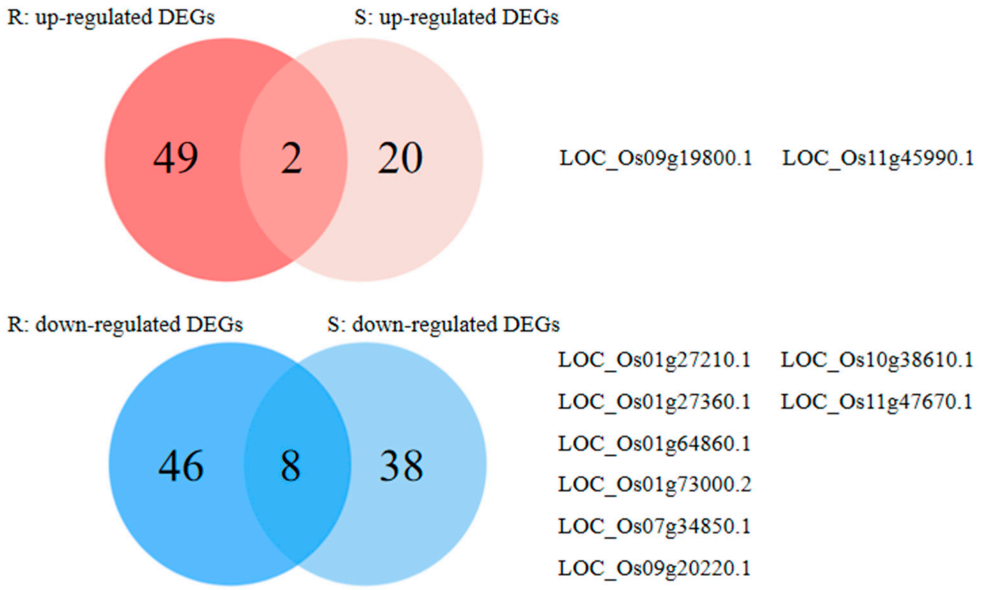

Figure S3. DEGs aligned to post-translational modification, protein turnover, and chaperones classifications.

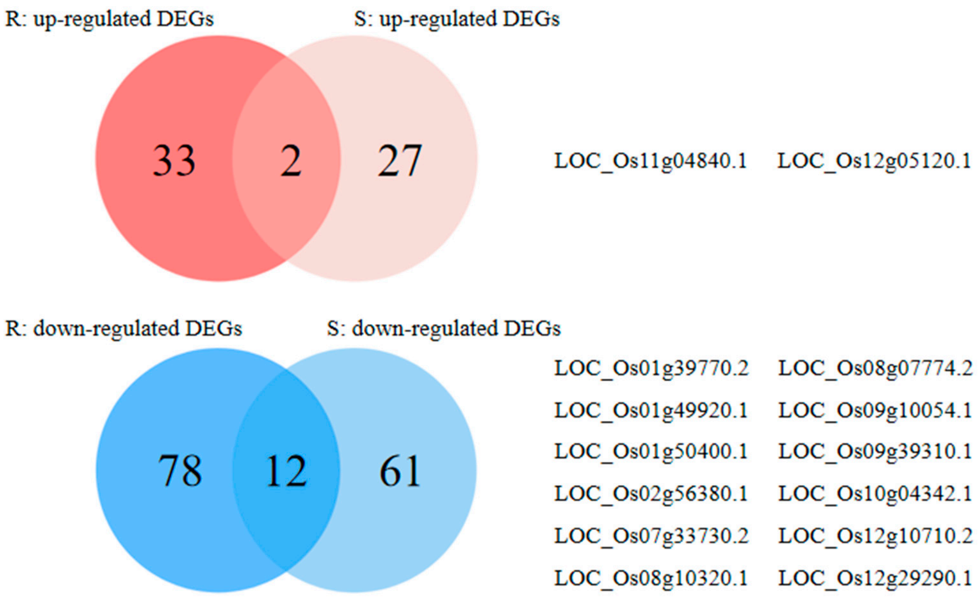

Figure S4. DEGs aligned to signal transduction mechanisms classifications.
